# Assessment of Anatomical Morphology of the Ileocecal Junction and Ileocecal Valve by Dissection and Endoscopy

**DOI:** 10.7759/cureus.61974

**Published:** 2024-06-08

**Authors:** Caroline Manoj, Yogapriya V, Patrick Paul

**Affiliations:** 1 General Internal Medicine, The Royal Wolverhampton NHS Trust, Wolverhampton, GBR; 2 Biochemistry, Madha Medical College and Research Institute, Chennai, IND; 3 Medicine, Edwin Eye Care, Wolverhampton, GBR

**Keywords:** ileoscopy, endoscopy, dissection, ileocaecal junction, ileocaecal valve

## Abstract

Introduction: The ileocecal valve (ICV) guards the opening of the ileocecal junction (ICJ) and acts as a mechanical barrier to prevent the reflux of material from the colon into the ileum. The morphology of the ICV noted in living is different from that of cadavers. The ICV is better studied in the living by endoscopy. A study of variation in the gross anatomy of the ICV can help determine the factors responsible for its competency.

Materials and methods: A descriptive study was conducted on a total of 85 (N) patients each in two groups over a period of two years. Group I: 85 specimens of the ileocecal region obtained during dissection; Group 2: 85 patients undergoing colonoscopic study undertaken in the Department of Medical Gastroenterology. In the dissection method, the apparently normal ICJ was inspected, photographed, and measured using Vernier calipers. In the colonoscopy method, the procedure was observed and while observing ICV, photos were taken. These photographs were later compared with other photographs in the literature to identify the morphology of ICV. The age, gender, location, and morphology of ICV were represented in percentile, and qualitative variables were analyzed by Pearson correlation coefficient.

Results: Out of 85 participants in Group A, 80% (68) were males and 20% (17) were females; in Group B, 58% (49) were males, and the remaining 42% (36) were females. In the dissection method out of 85 (N) patients, 98% had reverse S type terminal ileum, correlation of diameter of ICV with age p=0.003 and correlation of morphology of ICV with age p=0.006, and was statistically significant. In the colonoscopy method, 58% were males and 22% of them were 31-40 years. In 49% of patients, ICV was viewed from the left lateral position. The correlation of success of ileoscopy with age (p=0.608), gender (p=0.896), the position of the patient (p=0.236), and morphology of ICV (p=0.631) was not statistically significant.

Conclusion: There were no age-related changes observed regarding the morphology of the ICJ. It was found that as age increases diameter of ICV increases. The success of ileoscopy was highest in 31-40 years of age.

## Introduction

The ileocecal valve (ICV) is present at the junction of the small and large intestines. It guards the opening of the ileocecal junction (ICJ) and acts as a mechanical barrier to prevent the reflux of material from the colon into the ileum. ICV functions not just as a passive valve but also as a sphincter as the ileal papilla protrudes into the cecum [1]. The competency of ICV depends on mesenteric and other tenia, external ligaments attached to the ICJ, venous cushions in the submucosa of the terminal ileum, ileal papilla, and intussusception of terminal ileum into the cecum [2]. ICV promotes antegrade intestinal transport and regulates intestinal transit and is thus referred to as the “intestinal stomach,” which stores the residue and empties it into the colon. When ICV is resected, bacterial overgrowth occurs in the small intestine with inflammation of the ileal mucosa, leading to short bowel syndrome [3]. The type of ICV can vary based on the position of the patient, whether prone or supine. The morphology of the ICV noted in living is different from that of cadavers; The bilabial type of ICV is common in living, whereas the classic type of ICV is more common in cadavers. The study of morphology in ICV has a crucial impact on clinical medicine. 

ICV can be better studied in the living by endoscopy and is a vital diagnostic tool. It is used to diagnose conditions such as altered bowel habits, GI bleeding, chronic abdominal pain, and colorectal cancer. Surgical recreation of artificial ICJ is not very successful as the focus is on recreating a valve and the sphincteric mechanism is overlooked [4]. The main aim of this current study was to find the prevalence of the classical types of ICJ and ICV among cadavers by dissection, study the morphology of ICV in the living by endoscopy, and study the age-related changes in ICJ and ICV.

## Materials and methods

This study was a descriptive study conducted in the Department and Government Medical College, Thiruvananthapuram. The current study was conducted as thesis research for partial fulfillment of the MD degree requirement as per Kerala University of Health Sciences, Kerala. The study was conducted after obtaining ethical approval from the Institutional Ethical Committee. Informed consent was obtained from relatives of deceased patients and patients undergoing colonoscopy.

**Figure 1:**
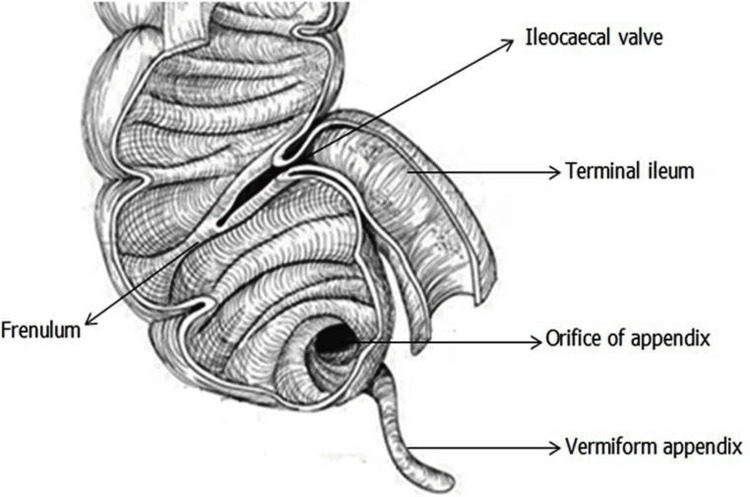
Morphology of ICV and its anatomical landmarks ICV, ileocecal valve

This study was conducted on a sample size of 85 (N) patients in two groups for over two years. Group I: 85 (N) specimens of the ileocecal region obtained during dissection in the Department of Anatomy and during autopsy in the Department of Forensic Medicine and Department of Pathology, Government Medical College, Thiruvananthapuram. Group 2: 85 (N) patients undergoing a colonoscopic study in the Department of Medical Gastroenterology, Government Medical College, Thiruvananthapuram. Inclusion criteria include cadavers with normal ICJ and ICV from autopsy cases of all age groups performed within six hours including normal fetal autopsy, and patients with normal ICV on colonoscopy were included in the study (Figure 1). Furthermore, autopsy cases brought six hours after death or with abdominal injuries or burns and patients undergoing colonoscopy with pathologies of the terminal ileum or large intestine with involvement of ICJ and ICV and right ileac fossa tumor with altered anatomy of ICJ were excluded from the story.

In the dissection method, the apparently normal ICJ was inspected and photographed. The ileocecal area was removed en bloc after sectioning of the ascending colon and terminal ileum. The specimens were opened by incision from the upper cecum along its lateral aspect and the ICV was then measured using a Vernier caliper or thread and high-resolution photographs were taken. The incision was extended in the coronal plane to reach the medial wall of the cecum then the ICV and terminal ileal loop were bisected. Further measurements and photographs were obtained from the posterior half of the specimen. In the colonoscopy method, the procedure was observed and while observing ICV, photos were taken. These photographs were later compared with other photographs in the literature to identify the morphology of ICV. Along with this, ileal intubation was visualized, and its success was recorded and compared to previous studies. The values obtained were entered into an Excel sheet and submitted for statistical analysis. Data analysis was done using appropriate statistical software (SPSS version 15). Quantitative variables were analyzed using mean and standard deviation. The age, gender, location, and morphology of ICV were represented in percentile, and qualitative variables were analyzed by Pearson correlation coefficient, and a P-value of <0.05 was considered as statistically significant.

## Results

The results of the study were broadly grouped into findings by dissection method and colonoscopy. Out of 85 participants in Group A, 80% (68) were males and 20% (17) were females; and in Group B, 58% (49) were males and the remaining 42% (36) were females (Table 1). 

**Table 1:** Distribution of gender by dissection method and colonoscopy

Gender, N=85 (100%)		
Method	By dissection	By colonoscopy
Group A: male	68 (80%)	49 (58%)
Group B: female	17 (20%)	36 (42%)

**Figure 2:**
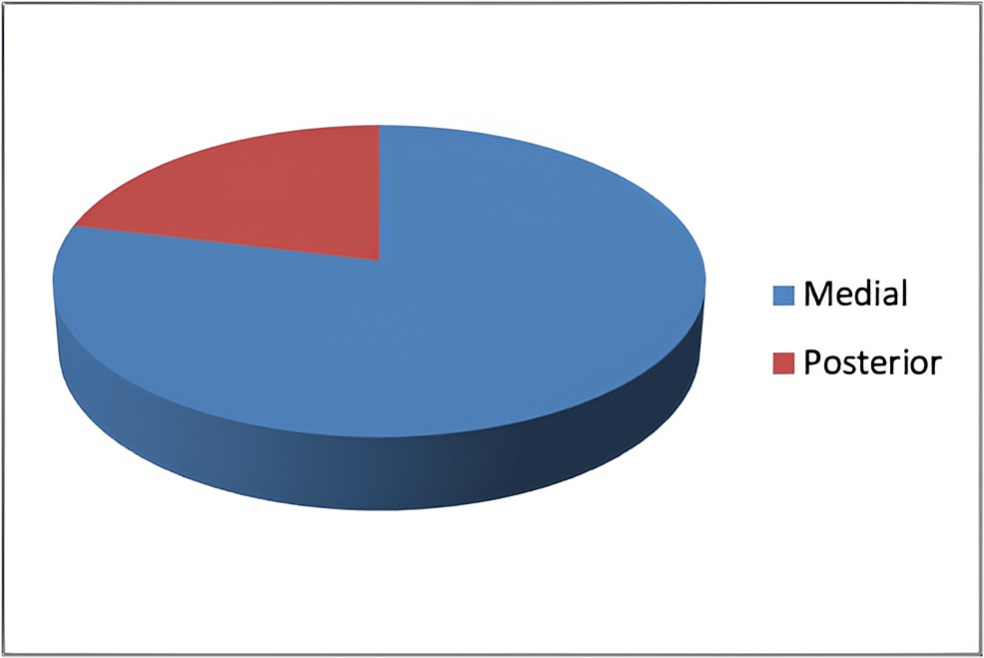
Pie chart showing an aspect of the cecum receiving terminal ileum

In the dissection method, the autopsy specimen was obtained in the age range of the first decade to the ninth decade. The majority of autopsies obtained were in the age range of 41-50 years (19%), followed by 21-20 years (18%), and was the least common in the age range of above 81 years (2%) followed by 4% (3) cases in the first decade. Out of 85 specimens analyzed by dissection method, 98% had reverse S type of terminal ileum and only 2% had horizontal type of approach of terminal ileum to cecum. In 79% of the specimens, the terminal ileum entered the medial aspect of the cecum and in 21%, the terminal ileum entered the posterior aspect of the cecum (Figure 2). 

**Figure 3:**
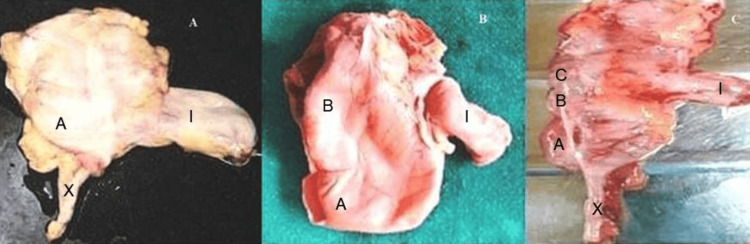
Dissected specimen showing the level of ICV in relation to cecal haustration A, 1 level haustration; B, 2 level haustration; C, 3 level haustration; l, ileum; x, appendix ICV, ileocecal valve

Out of 85 autopsy specimens, in 67%, the terminal ileum entered the cecum at the level of first haustration, and in 32%, the terminal ileum entered the cecum at the level of second haustration, and in the remaining 1%, terminal ileum entered the cecum at the level of third haustration (Figure 3). 

The mean measurement of ICV in 68 males was as follows: projection of the superior lip was 24.2 mm, projection of inferior lip was 22.8 mm, the diameter of ICV was 18.5 mm, and distance of appendicular orifice from ICV was 26.4 mm. In 17 females, the mean measurement of ICV was as follows: projection of superior lip was 22.8 mm, projection of inferior lip was 27.9 mm, the diameter of ICV was 19.5 mm, and the distance of appendicular orifice from ICV was 29.4 mm. Out of 85 specimens of ICV, type 1 was seen in 32%; type IIA in 4%; type IIB in 9%; type IIC in 34%; type III in 3%; type IVA, type IVB, and type V in 1%, papillary in 7%, and lipomatous in 8%. 

**Figure 4:**
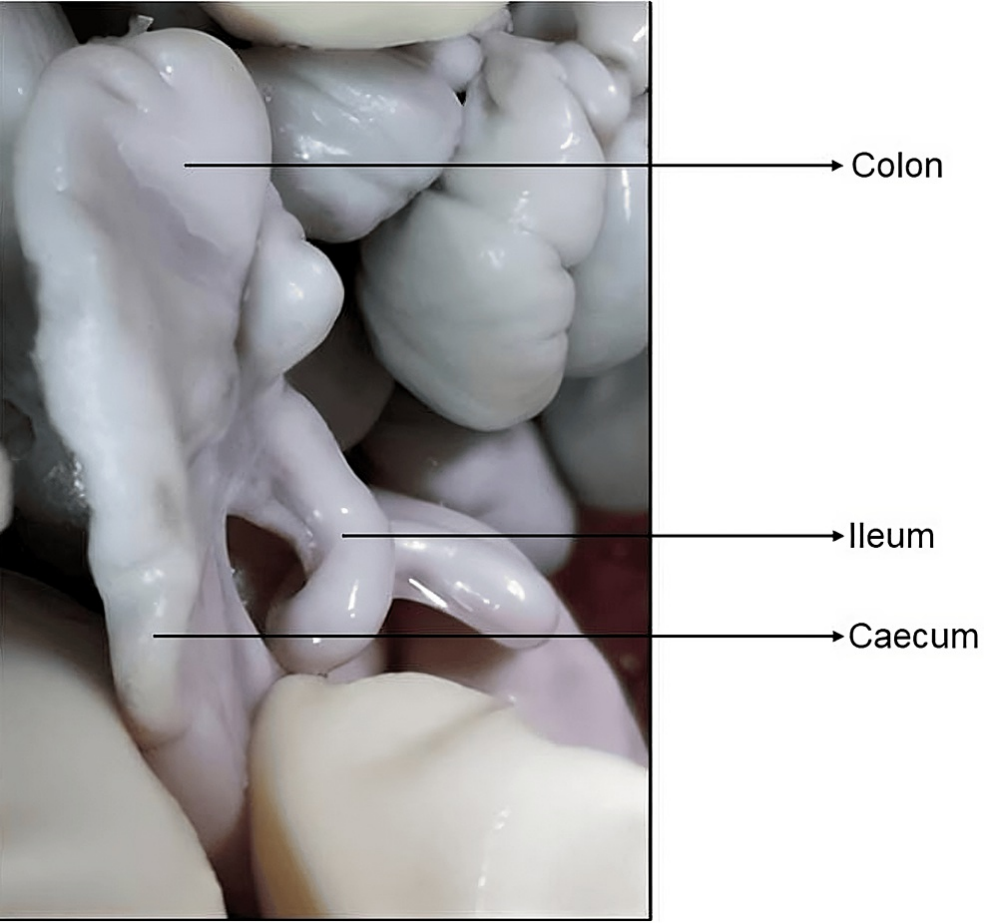
Reverse S type of ICJ seen in fetal autopsy ICJ, ileocecal junction

**Figure 5:**
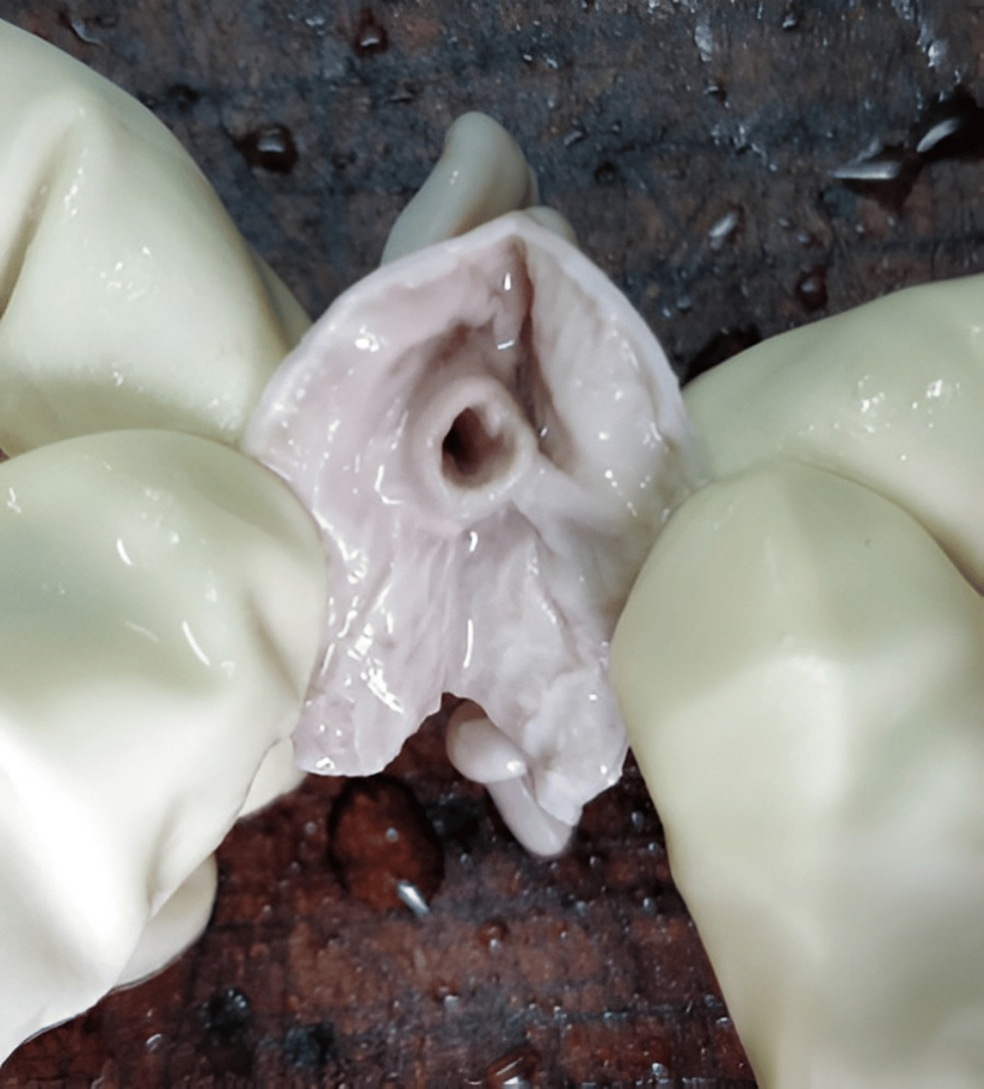
Papillary type of ICV seen in fetal autopsy ICV, ileocecal valve

Three fetal autopsies included in this study were male, and their gestational age was 30 weeks, 35 weeks, and one day post-natal, and the diameter of ICV was 0.4 mm, 0.5 mm, and 5 mm, respectively. The cause of death in two intrauterine fetuses was suspected congenital anomaly, and one post-natal death was due to infection. All fetal autopsies had reverse S type of ICJ in two cases, the terminal ileum entered anterior aspect of the cecum at the level of first haustration and in one case, the terminal ileum entered the medial aspect of the cecum at the level of second haustration (Figures 4, 5). 

**Figure 6:**
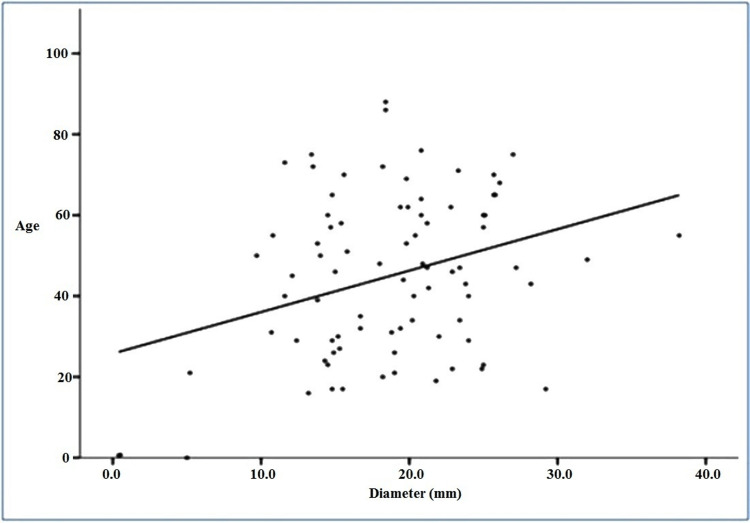
Correlation between age and diameter of ICV ICV, ileocecal valve

We correlated the diameter of ICV with the age of the patient using Pearson correlation and found that the P-value was 0.003. This shows that there was a positive correlation between age and the diameter of the ICV, which means that as age increases, the diameter of the ICV increases (Figure 6). 

We correlated the association of morphology of ICV with age and found that the P-value was 0.006. Correlation between age and morphology showed a P-value of <0.05 (Table 2). 

**Table 2:** Correlation between age and morphology

Morphology	N (85)	Age		P-value
		Mean	SD	
Type I	27	49.9	18.4	0.006
Type IIA	3	22.0	1.0	
Type IIB	8	58.9	14.1	
Type IIC	29	46.1	19.8	
Type III	2	38.0	5.7	
Type IVA	1	47.0	-	
Type IVB	1	17.0	-	
Type V	1	50.0	-	
Papillary	6	19.9	23.5	
Lipomatous	7	41.6	13.5	

**Figure 7:**
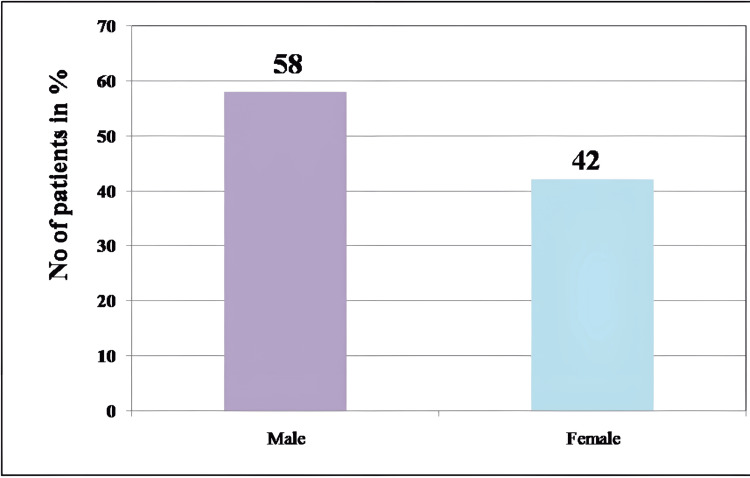
Bar graph showing the distribution of gender (%) in patients undergoing colonoscopy

Colonoscopies of 85 patients were observed, and the photographic images were studied. Of this, 58% (49) were males and 42% (36) were females (Figure 7). 

**Figure 8:**
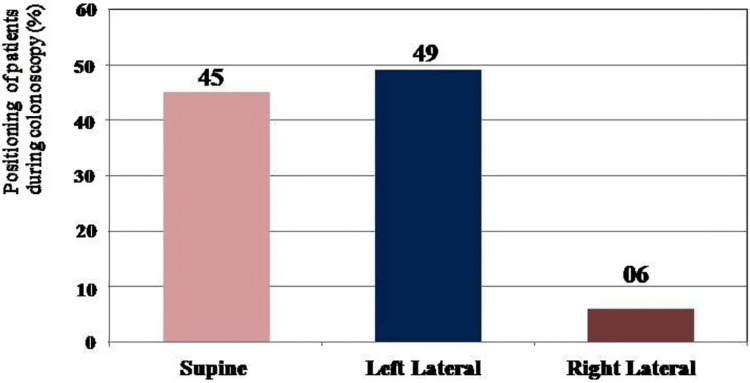
Bar graph showing the positioning of the patient (%) during colonoscopy

Patients undergoing colonoscopy were in the age range of ≤20 to ≥70 years. The majority of colonoscopy images (22%) were seen in the age range of 31-40 years and 51-60 years, followed by 41-50 years in 17% and least seen in 21-30 years (5%), followed by 7% in ≥70 years of age. Out of 85 patients, colonoscopy was indicated for abdominal pain in 15%, bleeding PR in 25%, mass or carcinoma in 21%, dyspepsia in 5%, altered bowel habits in 11%, inflammatory bowel disease in 13%, weight loss in 1%, and other symptoms in 9%. Out of 85 patients, the ICV was visualized with coloscopy in the supine position in 45%, left lateral positioning in 49%, and right lateral position in 6% of the subjects (Figure 8). 

Morphological type of ICV at colonoscopy was double bulge papillary type in 34%, bilabial in 33%, single bulge papillary in 21%, and volcanic in 12% of patients. Ileoscopy was successfully performed in 66% of subjects, whereas could not be done in 34% of subjects. 

We correlated the success of ileoscopy with age, gender, the position of the patient, and morphology of ICV with the success of ileoscopy and found that the success rate of ileoscopy was highest in the age range of 31-40 years and least successful in the age range of 61-70 years and above 70 years. The P-value noted was 0.608 suggesting that there was no correlation between age and success of ileoscopy (Table 3). 

**Table 3:** Correlation of age with success of ileoscopy, where χ2=4.508; df=6; P=0.608

Age	Ileoscopy				Total	
	Yes		No			
	N	%	N	%	N	%
≤20	5	56	4	44	9	100
21-30	3	75	1	25	4	100
31-40	15	79	4	21	19	100
41-50	10	71	4	29	14	100
51-60	13	68	6	32	19	100
61-70	7	50	7	50	14	100
71+	3	50	3	50	6	100
Total	56	66	29	34	85	100

Females showed a higher success rate of ileoscopy when compared to males. But the P-value was 0.896 suggesting no correlation between gender and the success of ileoscopy (Table 4). 

**Table 4:** Correlation of gender with success of ileoscopy, where χ2=0.017; df=1; P=0.896

Sex	Ileoscopy				Total	
	Yes		No			
	N	%	N	%	N	%
Male	32	65	17	35	49	100
Female	24	67	12	33	36	100
Total	56	66	29	34	85	100

The success of ileoscopy was highest when the patient was in the right lateral position and least when the patient was in the left lateral position. As the P-value was >0.05, there is no correlation between the position of the patient and the success of the ileoscopy (Table 5). 

**Table 5:** Correlation of position of the patient with success of ileoscopy, where χ2=2.885; df=2; P=0.236

Position	Ileoscopy				Total	
	Yes		No			
	N	%	N	%	N	%
Supine	25	66	13	34	38	100
Left lateral	26	62	16	38	42	100
Right lateral	5	100	0	0	5	100
Total	56	66	29	34	85	100

Single bulge type of papillary ICV had the highest rate of successful ileoscopy while the bilabial type had the highest rate of unsuccessful ileoscopy. The P-value was >0.05, which suggests a lack of correlation between the morphology of ICV and the success of ileoscopy (Table 6). 

**Table 6:** Correlation of morphology of ICV with success of ileoscopy, where χ2=1.729; df=3; P=0.631 ICV, ileocecal valve

Morphology of ICV	Ileoscopy				Total	
	Yes		No			
	N	%	N	%	N	%
Bilabial	17	61	11	39	28	100
Papillary: single bulge	14	78	4	22	18	100
Papillary: double bulge	18	62	11	38	29	100
Volcanic	7	70	3	30	10	100
Total	56	66	29	34	85	100

**Figure 9:**
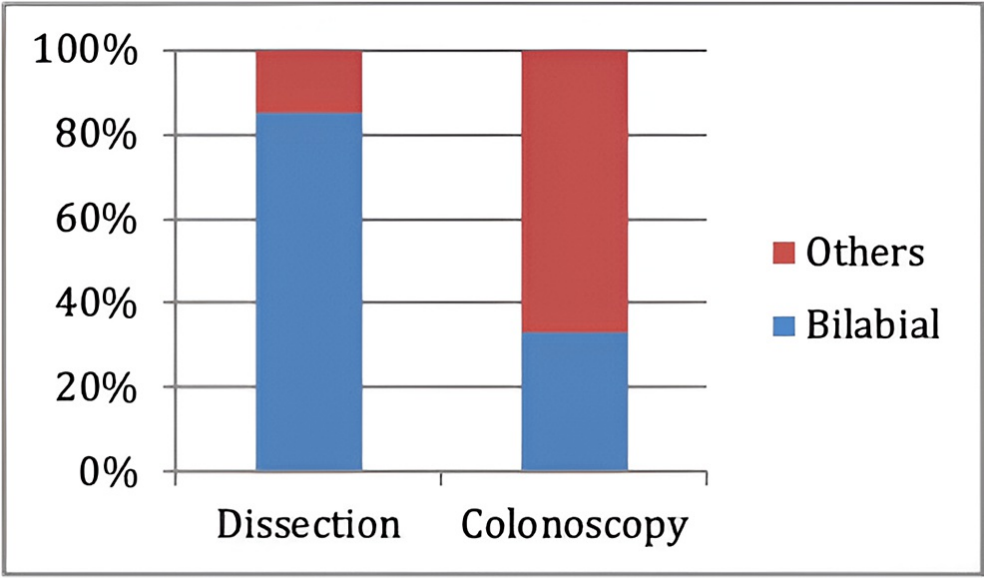
Prevalence of bilabial type of ICV in dissected specimens and patients undergoing colonoscopy

The most seen ICV type in the dissection method was the bilabial type of ICV in 85% of specimens while the remaining 15% comprised other types of ICV. Whereas in the colonoscopy method, 33% of subjects showed the bilabial type of ICV and the remaining 67% comprised other types of ICV (Figure 9). 

## Discussion

The ICV was discovered by Swiss anatomist, botanist, and physician Caspar Bauhin in 1579 hence it is called the Bauhin valve. ICV prolongs the intestinal transit time thereby enhancing the absorption of nutrients, fluids, and electrolytes. It was also described by Dutch physician Nicolas Tulp hence it is also called the Tulp valve. Varoli in 1573 described this valve as the "Operculum Ilei" and defined its physiological attributes [5]. Keith A in 1912 proposed a division of the cecum into three parts, namely, the cecal colon above the ICV and retinacular bands, the cecum proper below the ICV, and an apical part or appendicular part with a narrow lumen [6]. ICV is formed by two folds of mucous membrane at the opening of the ileum into the large intestine known as the upper lip and lower lip; the upper lip is longer than the lower lip [7]. The diameter of normal ICV is 2.5 cm to 3.5 cm, the normal thickness of the upper and lower lip of ICV is 0.3 cm to 3 cm, and the projections of lips into the cecal lumen are normally 0.6 cm. Normal ICV can have different appearances based on cecal mobility (based on the laxity of mesenteric attachments) and distension, whether the valve is open or not and also because of inherent variability in its morphology. During colonoscopy, ICV is used to identify the cecum based on the direction of curvature at the orifice. It is suggested that intubation of ICV during colonoscopy helps in the evaluation of the distal or lowest part of the ileum [8].

The objective of this study was to find out the prevalence of the classical type of ICJ and ICV among cadavers by dissection and to study the morphology of ICV in living by endoscopy. This study also explores ICJ and the positional relationship between terminal ileum and cecum. In the present study, we found that in 85 subjects, the approach of terminal ileum to cecum was predominantly reverse S type rather than horizontal type. The findings of our study are similar to the findings of Hunter R (1934), Oppenheimer A (1933), and Fleischner (1950), who found that a reverse S-shaped terminal ileum was noted in 95% of cases than horizontal type. The ileum approaching the cecum from above was rarely seen [9-11]. The terminal ileum entered the medial aspect of the cecum in most of the subjects in this study and is like the previous findings of Oppenheimer A, 1933; Fleischner, 1950; and El-Amin, 2003; who also reported that ICV was located most on the medial wall followed by lateral and least commonly on the posterior wall of cecum [10-13]. The terminal ileum entered the cecum at the level of first haustration in the majority of cases, followed by the level of second haustration, and third haustration in the least subjects. These findings of our study contradict the findings of El-Amin, 2003, who found that ICV is located most at the level of second haustration, followed by first haustration, and least at the level of third haustration [13].

As an additional finding in the present study, we found that the mean length of the medial frenulum was 6 cm, the mean height of the medial frenulum was 1 cm, mean length of lateral frenulum was 6.5 cm, mean height of the lateral frenulum was 1 cm, mean length of the superior lip was 4 cm, mean length of superior lip projection was 3 cm, mean height of the superior lip was 1.5 cm, mean length of the inferior lip was 4 cm, mean length of the inferior lip projection was 2.5 cm, the mean height of the inferior lip was 1 cm, average diameter was 1.9 cm, and mean distance of the ICV from appendicular orifice was 3.1 cm. The findings of the present study corroborate with the observations of Rutherford A (1914), Rutherford A (1926), Buirge R (1943), and Ulin A (1956) [12-16]. However, our findings contradict the findings of Kraus O (1912) and Oppenheimer A (1933) who stated that the upper lip of ICV is much longer than the lower lip and the diameter of ICV is around 0.8 cm [7,10].

The most predominantly seen morphological type of ICV in our study was type II C, followed by classical and the least common types of ICV were type IVA, IVB, and V. This finding of the present study was like the findings of Kellogg (1914) and Short A (1919), who found that the most common type of ICV was valvular with variations followed by papillary and lipomatous [8,17]. Conversely, our findings contradict the findings of Buirge (1943) and Awapittaya (2007) who observed that the most common morphological type of ICV was type I, and type IIC was the least common type [16,18]. In all three cases of fetal autopsy studied, ICJ had reverse-S morphology and the most common morphological type of ICV was papillary type. These finding of ours agrees with the findings of Beattie J (1924), Perrin (1921), R Balli (1939), Bogers J (1993), and Pollard (2012) [19-23]. In this study, the correlation of ileoscopy with age showed the highest rate of success in the fourth decade of life, in females, in the right lateral position. The findings of the study suggest that the diameter of ICV increases with an increase in age and were highly statistically significant and so was the association of age and morphology of ICV. However, an association of age, gender, the position of the patient, and morphology of ICV did not show any association with the success of ileoscopy and was not statistically significant. To the best of our knowledge, we did not come across any literature, which has correlated these findings.

Out of 85 patients, the majority were positioned in the left lateral position during visualization of ICV at colonoscopy. These findings are like the findings of Iacopini (2006) and Ahammed (2014) [24,25]. In our study, we found that there was no statistical association between the success of ileoscopy and gender, age of the patient, BMI, position of patient, or morphology of ICV. These findings were like the findings of Iacopini (2006) [24]. The most commonly seen morphological type of ICV was double bulge papillary type ICV and the volcanic type was the least common and is like the studies of Ahammed S (2014), Williams (1973), and Silva (2007) [25-27]. This finding is contradictory to the findings of Iacopini (2006) and Regge D (2005) who highlight the difference in morphological types of ICV among the South-Indian population compared to the Western population (Italy) [24,28]. Competency in ICV intubation is achieved by the endoscopist after 50 procedures and contrasts the findings of Ahammed S (2014) who found that younger age, supine posture, and morphological type of ICV determine the success of ileal intubation [25]. We found that the bilabial type of ICV was the most prevalent type of ICV in dissection specimens than patients undergoing colonoscopy and is consistent with the findings of Rutherford A (1926) and Ulin A (1956). The appearance of ICV changes rapidly after death and becomes more patulous after death. The circular mass relaxes due to the loss of tone of circular muscles of the ICV and the orifice becomes slit-like post-mortem [15,29].

There were a few limitations encountered in the present study; the majority of the cases that were available for both dissection and colonoscopy were males and the sample size of females was less. Also, measurements of the ICV could not be taken at colonoscopy due to a lack of the appropriate infrastructure. The age-related changes in diameter and morphological type of ICV are two of the most significant findings in the present study and more studies with a larger sample size and equal distribution of gender in a new venture may better describe the morphology of ICV and will be extremely helpful for the clinician during the procedure of colonoscopy and ileoscopy. We, however, recommend that this study when conducted with a larger sample size as randomized clinical trials with equal distribution of males and females and the latest equipment may yield better results.

## Conclusions

Morphology of the ICJ is described as the positional relationship of the terminal ileum to the cecum in the present study, and the most seen approach of terminal ileum to cecum was reverse S type. Terminal ileum entered the cecum from the medial aspect most commonly and at the level of the first haustration. The most common morphological type of ICV seen in the dissection method was Type IIC and in the endoscopy method, it was the double-bulge papillary type. There were no age-related changes observed regarding the morphology of the ICJ. It was found that as age increases, the diameter of ICV increases. The success of ileoscopy was highest in 31-40 years of age.
